# Novel Contact Lenses Embedded with Drug-Loaded Zwitterionic Nanogels for Extended Ophthalmic Drug Delivery

**DOI:** 10.3390/nano11092328

**Published:** 2021-09-07

**Authors:** Zhao Wang, Xinhua Li, Xiaojuan Zhang, Ruilong Sheng, Qing Lin, Wenli Song, Lingyun Hao

**Affiliations:** 1 School of Material Engineering, Jinling Institute of Technology, Nanjing 211169, China; lixinhua@jit.edu.cn (X.L.); xixi@jit.edu.cn (X.Z.); lnqing@jit.edu.cn (Q.L.); wlsong@jit.edu.cn (W.S.); 2 Nanjing Key Laboratory of Optometric Materials and Technology, Nanjing 211169, China; 3 CQM—Centro de Química da Madeira, Campus da Penteada, Universidade da Madeira, 9000-390 Funchal, Portugal; ruilong.sheng@staff.uma.pt

**Keywords:** contact lenses, nanogels, ophthalmic drug delivery, sustained release

## Abstract

Therapeutic ophthalmic contact lenses with prolonged drug release and improved bioavailability have been developed to circumvent tedious eye drop instillation. In this work, zwitterionic nanogels based on poly(sulfobetaine methacrylate) (PSBMA) were easily fabricated by one-step reflux-precipitation polymerization, with the advantages of being surfactant-free and morphology controlled. Then, the ophthalmic drug levofloxacin (LEV) was encapsulated into the nanogels. A set of contact lenses with varied nanogel-loading content was fabricated by the cast molding method, with the drug-loaded nanogels dispersed in pre-monomer solutions composed of 2-hydroxyethyl methacrylate (HEMA) and N-vinyl-2-pyrrolidone (NVP). The structure, surface morphology, water contact angle (WCA), equilibrium water content (EWC), transmittance, and mechanical properties of the contact lenses were subsequently investigated, and in vitro drug release and biocompatibility were further evaluated. As a result, the optimized contact lens with nanogel-loading content of 8 wt% could sustainably deliver LEV for ten days, with critical lens properties within the range of recommended values for commercial contact lenses. Moreover, cell viability assays revealed that the prepared contact lenses were cytocompatible, suggesting their significant potential as an alternative to traditional eye drops or ointment formulations for long-term oculopathy treatment.

## 1. Introduction

With the rapid growth in the aging population and the prevalence of electronic products, the number of people afflicted with ocular diseases (cataracts, dry eye syndrome, infections, glaucoma, age-related macular degeneration, etc.) is increasing year by year [1]. Topical delivery (i.e., eye drops and ointments), is the preferred route of administration and accounts for more than 90% of ophthalmic formulations on the market [2]. However, reflex blinking, tear turnover and low permeability of the corneal tissue, etc., constrain this topical approach, thus providing a nonoptimal dosage with only approximately 3–5% bioavailability [3,4]. An ideal administration method needs to be biologically well tolerated, easy to administer, and have no significant adverse effects on normal ocular functions, such as vision or blinking.

Contact lenses have been widely used by millions of people worldwide for decades for vision correction [5]. They are biocompatible, safe, durable and comfortable, thus providing a new route for ophthalmic drug delivery [6]. Numerous animal studies demonstrated that ophthalmic medications delivered by therapeutic contact lenses could prolong the time a drug stays on the cornea and increase its bioavailability by at least 50%, compared with eye-drop formulations [1,7]. A conventional soaking method is the most straightforward and cost-effective way to load drugs into contact lenses [8]. However, limited drug-loading and burst release are prevalent problems, with complete drug release from the lens within a few hours. Recently, some innovative strategies, such as in situ creation of a vitamin E barrier [9], molecular imprinting technology [10], drug-laden nanoparticles, the introduction of ionic or ‘host-guest’ interactions [11] and the incorporation of drug-loaded polymer films [12] have been reported to achieve the continuous release of drugs [13,14,15,16,17,18]. Although great efforts have been achieved in drug-eluting contact lenses, however, the critical lens properties, such as optical transparency, ion and oxygen permeability, mechanical durability, serialization and storage stability, as well as patient and practitioner acceptance have yet to be addressed [19].

Colloidal nanoparticles (nanogels, liposomes, micelles, microemulsions, etc.) have attracted great attention as drug delivery carriers because of their efficient drug loading and sustained-release properties [20,21]. Incorporating drug-loaded nanocarriers into hydrogels will prolong the drug-release time [22,23], for drugs need to release from the nanoparticles and then diffuse through the hydrogel matrix to reach the action site. Moreover, encapsulation of drugs in colloidal nanoparticles can prevent drug degradation by ocular enzymes. The drug release rate and targeted therapy can be further improved by the effective design of delivery vehicles. The mPEG-PCL micelle-laden contact lens prepared by Tang et al. for the treatment of glaucoma achieved the sustained release of timolol and latanoprost in tears for more than four days [24]. Akbari et al. [25] recently prepared hyaluronic acid (HA)-loaded chitosan (CS) nanoparticles, which were dispersed in a ring shape poly(vinyl alcohol) (PVA) hydrogel to form the ring-implanted PVA contact lens. Results showed a sustained release of HA from the contact lens for treatment of dry eye syndrome for up to 14 days. A special contact lens made of Bri@LDH/Thermogel composite drug delivery system (DDS) developed by Sun et al. [26] showed sustained brimonidine release for up to 168 h, and effectively relieved intraocular pressure (IOP). Furthermore, in cyclosporine-loaded Eudragit S100 (pH-sensitive) nanoparticle-laden contact lenses designed by Maulvi et al. [27], in vitro drug-release experiments showed no significant leaching of the drug during sterilization and the storage period (pH 6.5). At the same time, accelerated drug release was achieved in a tear environment (pH 7.4) because of the dissolution of Eudragit S100. Kumar et al. [28] also reported extended levobunolol release from pH-sensitive Eudragit nanoparticle-laden contact lenses for glaucoma therapy. However, the natural aggregation of nanoparticles reduced the optical transparency of contact lenses, and the decreased water content caused by the incorporation of nanoparticles reduced the wear comfort of the contact lenses.

Nanogels are defined as hydrogel nanoparticles with sizes in the range of 1–1000 nm formed by physical or chemical cross-linking [29]. Nanogels are hydrophilic, flexible, highly water-absorbent, biocompatible, etc., and their size, charge, porosity, hydrophilicity, flexibility and degradability can be adjusted by changing the chemical composition of nanogels. They can load drugs, proteins or DNA efficiently via salt bridges, hydrogen bonds and/or hydrophobic interactions, and their large surface area facilitates the conjugation of multivalent biomolecules. In particular, because of the unique chemical cross-linked structure and large specific surface area, nanogels have lower dissociation/degradation rates and better water retention capacity, which results in higher colloidal stability than in polymeric micelles. To the best of our knowledge, there are no reports on nanogel-embedded contact lenses for extended drug delivery.

**Scheme 1:**
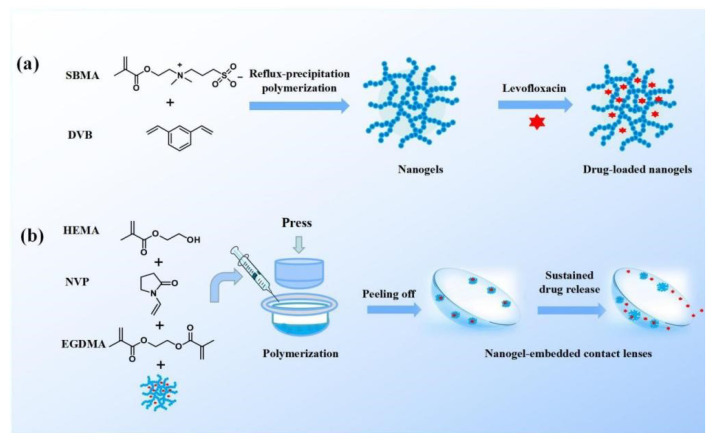
The reflux-precipitation polymerization of nanogels and preparation of drug-loaded PSBMA nanogels (**a**) and the fabrication of the nanogel-embedded contact lenses (**b**).

Zwitterionic polymers interact with water molecules via both electrostatic forces and hydrogen bonding to form a hydration layer [30]. Accordingly, zwitterionic nanogels possess high water-binding capability and anti-protein-fouling performance [31]. Thus far, researchers have introduced zwitterionic polymers into the hydrogel matrix to suppress the protein/bacteria adsorption of contact lenses. Likewise, Shen et al. [32] developed contact lenses with a surface modified with copolymers poly(3-trimethoxysilyl-propyl-methacrylate)-block-poly(2-methacryloyloxyethyl phosphorylcholine) (PMPS-*b*-PMPC), which showed significant surface-wettability improvement, protein adsorption resistance, and superior antibacterial adhesion properties. Additionally, some zwitterionic compounds, such as sulfobetaine methacrylate (SBMA), carboxymethyl betaine (CMB), etc., were used as comonomers and incorporated into the contact lens hydrogel matrix to improve anti-biofouling properties [33,34,35]. Herein, we prepared PSBMA nanogels by a convenient reflux-precipitation polymerization method [36], and the anti-inflammatory drug levofloxacin (LEV) was then incorporated into nanogels via hydrophobic and intermolecular interactions. Nanogel-embedded contact lenses were then prepared by cast molding method with the drug-loaded nanogels dispersed in pre-monomer solutions comprising HEMA and N-vinyl-2-pyrrolidone (NVP) for extended ophthalmic drug delivery (Scheme 1). The drug-release behaviors and other properties relevant to wear comfort, including transparency, hydrophilicity, swelling ability, mechanical properties and cytotoxicity were further characterized.

## 2. Materials and Methods

### 2.1. Materials

Hydroxyethyl methacrylate (HEMA, 99%), N-vinyl pyrrolidinone (NVP, 99%) and [2-(methacryloyloxy) ethyl] dimethyl-(3-sulfopropyl) ammonium hydroxide (SBMA, 97%) were purchased from Shanghai Macklin Biochemical Technology Co., Ltd. (Shanghai, China) and utilized as-received. Ethylene glycol dimethacrylate (EGDMA, 95%), divinylbenzene (DVB, 80%) and levofloxacin (LEV, 98%) were purchased from Shanghai Aladdin Biochemical Technology Co., Ltd. and utilized as received. N,N’-azobis (isobutyronitrile) (AIBN, 98%, Shanghai Macklin Biochemical Technology Co., Ltd.) was recrystallized twice from methanol prior to use. All other reagents and solvents were of analytical grade and used without further purification.

### 2.2. Preparation Process of PSBMA Nanogels

A series of PSBMA nanogels based on SBMA and divinyl benzene (DVB) were synthesized by reflux-precipitation polymerization with acetonitrile as the solvent, as referred to in the literature [37]. The preparation process was described as follows: SBMA and DVB (200 mg in all) and the initiator azobisisobutyronitrile (AIBN, 4 mg) were dissolved in 40 mL of acetonitrile in a dried 150 mL three-necked flask with the aid of ultrasonic agitation for 10 min. The flask was submerged in a 90 °C oil bath and reacted for 1 h. The obtained nanogels were separated by high-speed centrifugation, washed and purified by acetonitrile twice, alcohol twice and deionized water twice (centrifugation: 9000 rpm for 15 min). A series of PSBMA nanogels were prepared by changing the content of the SBMA monomer and DVB crosslinker, and the recipes are shown in Table 1.

### 2.3. Drug Loading

For LEV loading in nanogels, 10 mg of LEV was dissolved in 1 mL of glacial acetic acid aqueous solution (10 mM). Afterward, 50 mg of nanogel was added to the LEV solution, and the mixed solution was stirred for 24 h at room temperature. The mixed solution was dialyzed in deionized water for 48 h to form a drug-loaded nanogel aqueous solution. The resulting drug-loaded nanogels were lyophilized. The drug-loading levels were measured by UV-Vis spectrophotometer (Evolution 220, Thermo Fisher, Waltham, MA, USA) at an excitation wavelength of 292 nm after total drug elution by adding SDS (1 M). The drug-loading content (DLC) and drug-loading efficiency (DLE) were calculated according to a standard curve as following formulations:

(1) $$\mathrm{DLC}\left(\mathrm{wt}\%\right)=\frac{\mathrm{Weitht}\;\mathrm{of}\;\mathrm{loaded}\;\mathrm{drug}}{\mathrm{Weight}\;\mathrm{of}\;\mathrm{loaded}\;\mathrm{drug}\;\mathrm{and}\;\mathrm{polymers}}\times 100\%\;$$

(2) $$\mathrm{DLE}\left(\%\right)=\frac{\mathrm{Weight}\;\mathrm{of}\;\mathrm{loaded}\;\mathrm{drug}}{\mathrm{Weight}\;\mathrm{of}\;\mathrm{drug}\;\mathrm{in}\;\mathrm{feed}}\times 100\%$$

### 2.4. Hydrogel Preparation

The free contact lens and drug-loaded nanogel-embedded contact lenses were fabricated by the cast molding method via free radical polymerization. The basic materials for preparing hydrogels based on the comonomers of HEMA and NVP, the crosslinker of ethylene glycol dimethacrylate (EGDMA), the initiator of AIBN, and the drug-loaded nanogels are shown in Table 2. A colloidal dispersion (100 μL) was sandwiched between the upper mold and the bottom mold of a contact lens, with care to avoid bubbles. Then, the mold was set at 65 °C for 8 h for polymerization. Subsequently, the bottom mold was removed, and the solid-state contact lens with the attached upper mold was treated with 100 mL aqueous alcohol (20 wt%) to obtain a freestanding contact lens. The lens was then immersed in deionized water, which was replaced every 4 h for three consecutive days to remove free compounds. Finally, the clean lenses were immersed in deionized water before use. The drug-soaked contact lenses were manufactured by soaking free pHEMA lenses in 0.02 mM of LEV glacial acetic acid aqueous solution for 3 days.

### 2.5. Characterization

#### 2.5.1. Structure Characterization

The structures for the synthesized nanogels and contact lenses were characterized by Fourier transform infrared spectra (FTIR). FTIR spectra were obtained on a Nicolet iS5 spectrometer (Thermo Fisher) at room temperature with 32 scans spanning a spectral range of 4000–500 cm^−1^ and a resolution of 4.0 cm^−1^. The nanogels were compressed into KBr pellets, and the contact lenses were characterized directly by attenuated total reflection (ATR) mode.

#### 2.5.2. Particle Size Measurement

Particle sizes and zeta potentials of the nanogels were analyzed at 25 °C on a Zetasizer Nano ZS90 dynamic light scattering (DLS, Malvern, Worcestershire, UK) instrument equipped with a 4 mV He−Ne laser operating at λ = 633 nm and a fixed scattering angle of 90°. Each measurement was repeated three times, and the average value was accepted as the final result.

#### 2.5.3. Stability Characterization

PSBMA-4 was chosen as a representative to evaluate the stability of the PSBMA nanogels. First, the storage stability of nanogels was detected by monitoring the hydrodynamic size by DLS after incubation time (up to 30 days) in water at room temperature. Then, the size and size distribution of nanogels at a concentration of 1 mg mL^−1^ were measured after submersion in pH 7.4 phosphate buffer (PBS) with different concentrations of salt (0, 0.2, 0.3, 0.5 and 1 M) for 24 h.

#### 2.5.4. Morphological Observation

Morphologic evaluation of the nanogels and surface morphology of the contact lenses were conducted using field-emission scanning electron microscopy (FE-SEM) (SU8010, Hitachi, Tokyo, Japan). Briefly, a few drops of the previously prepared PSBMA-4 nanogel solution (1 mg/mL) were deposited onto a silicon wafer, followed by drying in a vacuum oven overnight. The prepared contact lenses were first placed in deionized water for several days to reach the swelling equilibrium, then lyophilized in a vacuum freeze dryer at −50 °C for 24 h before observation. All the samples were spattered with gold before observation.

#### 2.5.5. Transmittance

The optical properties of pHEMA contact lenses and nanogel-embedded contact lenses were measured via UV−Vis spectrophotometer in the range from 400 nm to 800 nm. Before the measurement, the hydrogels were soaked in water overnight for complete hydration. The sample was mounted in a quartz cuvette and placed in a spectrophotometer, and its transmittance was performed directly at 0.5 nm intervals.

#### 2.5.6. Water Contact Angle

The static water contact angles (WCA) of various contact lenses were measured according to the sessile drop method using a contact-angle analyzer (OCA 20, Dataphysics Instruments, Filderstadt, Germany). Briefly, the contact lens was soaked in deionized water overnight to reach the swelling equilibrium. After the free water on the surface of the hydrogel was wiped off gently by soft tissue paper, a drop of water (5 μL) was placed on the hydrogel surface. The contact angle was immediately (within 5 s) measured from the image of the water drop. For each type of contact lens, the average value calculated on three sample tests was used.

#### 2.5.7. Equilibrium Water Content

Equilibrium water content (EWC) of pHEMA contact lenses and nanogel-embedded contact lenses was measured using at least three lenses as a set to minimize error. First, after completely drying the fabricated lenses at 80 °C for 24 h, the weight was measured (W_dry_). Then, until the dried lenses were completely hydrated in water, the free water on the surfaces of the swollen lenses was gently wiped off by soft tissue paper, and the weight was measured (W_wet_). The EWC was calculated from Equation (3), and the experiment was repeated three times to obtain an average.

(3) $$\mathrm{EWC}=\frac{W_{\mathrm{wet}}-W_{\mathrm{dry}}}{W_{\mathrm{dry}}}\times 100\%$$

#### 2.5.8. Swelling Properties

The dried contact lenses (at least three pieces) were weighed (W_0_) and then placed in 15 mL of water, and the temperature was maintained at 37 °C. The lenses were removed every 0.5 h, and free water on the lenses was gently wiped off by soft tissue paper, then weighted W_t_. The samples were tested three times to obtain the average, and the degree of swelling was calculated as follows:

(4) $$\mathrm{Swelling}\;\mathrm{ratio}\left(\%\right)=\frac{W_{t}-W_{o}}{W_{o}}\times 100\%\;$$

#### 2.5.9. Mechanical Properties

For the mechanical properties test, hydrogel sheets, rather than samples in the shape of contact lenses, were fabricated. The fully hydrated hydrogels were sliced into rectangles (length × width × thickness = 20 mm × 10 mm × 0.5 mm) before measurement. Then, the tensile properties of various hydrogels fully hydrated in deionized water were measured using an electronic universal testing machine (EUTM, Instron 7540, Boston, MA, USA) at room temperature with the velocity of the tensile test fixed at 15 mm/min.

### 2.6. In Vitro Drug Release

The drug-release experiments were carried out by soaking drug-soaked contact lenses or nanogel-embedded contact lenses in 10 mL of PBS (10 mM, pH 7.4) at room temperature with simultaneous gentle shaking. At regular time intervals, 2 mL of release sample was removed, and 2 mL of fresh PBS was added to the release medium. The amount of drug released was calculated according to the calibration curve of LEV in PBS using a UV-Vis spectrophotometer (Evolution 220, Thermo Fisher) at an excitation wavelength of 292 nm. All the drug-release experiments were performed in triplicate for each time point. The drug-release profile for drug-loaded nanogels was performed using the dialysis bag method (molecular weight cut-off, 3500 Da). All the other procedures were the same as described above.

### 2.7. Cell Culture and Toxicity Test

The cytotoxicity of contact lenses was evaluated referring to ISO standard 10993-5. Mouse embryo fibroblast (3T3) cell lines were purchased from Cell Bank of the Chinese Academy of Sciences (Shanghai, China), and were cultured in Dulbecco’s Modified Eagle’s medium (DMEM) supplemented with 10% fetal bovine serum (FBS), penicillin (100 IU/mL) and streptomycin (100 μg/mL) at 37 °C with 5% CO_2_. Before measurement, the contact lenses were soaked in 75% aqueous alcohol for 4 h for sterilization, and subsequently washed with PBS three times. The cell viabilities were analyzed with the standard Cell Counting Kit-8 (CCK-8) assay. Briefly, the contact lenses were placed on the bottom surface of a 24-well tissue culture plate (TCP) at a density of 4 × 10^4^ cells per well, then 3T3 cells were sequentially seeded. After incubation for 1, 2, 3 and 4 days, the culture medium was replaced by fresh DMEM containing CCK-8 and incubated for another 1 h. Then, each sample was transferred into separate wells of a 96-well plate to determine the optical density (OD) value at 450 nm using a microplate reader (Epoch 2 Microplate Spectrophotometer, BioTek Instruments, Inc., Winooski, VT, USA) at an absorbance value of 450 nm. The final results were calculated with *n* = 5.

For cell morphology observation by confocal laser scanning microscope (CLSM), 3T3 cells were seeded onto a 35 mm glass-bottom dish with covered contact lenses at a density of 5 × 10^4^ cells per well. After incubating for 1, 2, 3 and 4 days, respectively, the culture medium was removed, and the contact lenses were washed by PBS three times. Afterward, the cells were stained with 10 μM calcein-AM solution for 20 min, and then fixed for 15 min with 4% paraformaldehyde aqueous solution at 37 °C. Finally, the stained 3T3 cells were observed by CLSM with excitation wavelengths of 499 nm and emission wavelengths of 515 nm, respectively.

## 3. Results

### 3.1. Preparation and Characterization of PSBMA Nanogels

**Figure 1:**
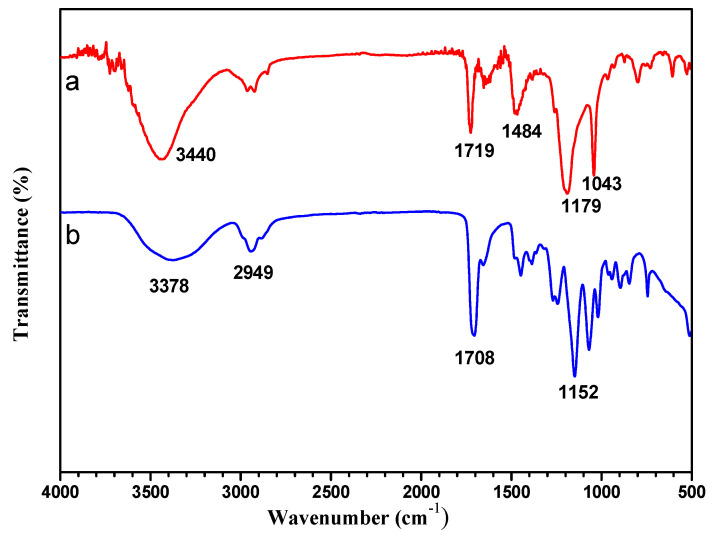
FTIR spectra of PSBMA nanogels (**a**) and pHEMA contact lens (**b**).

**Figure 2:**
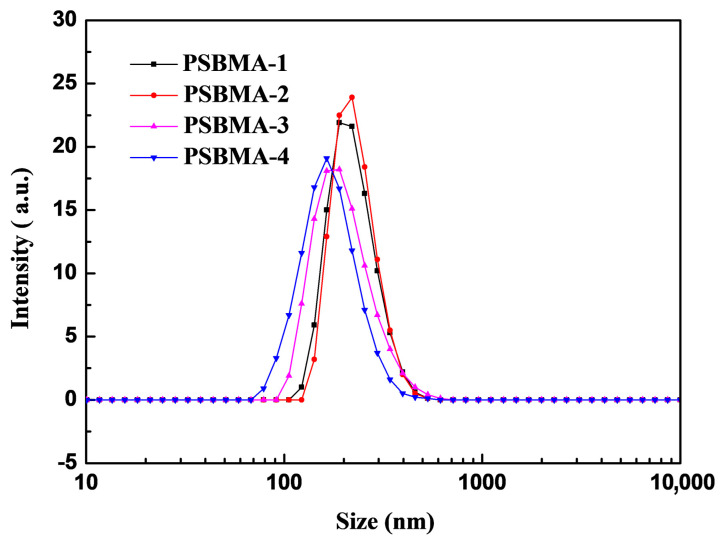
Size distributions for the series of PSBMA nanogels determined by DLS.

**Figure 3:**
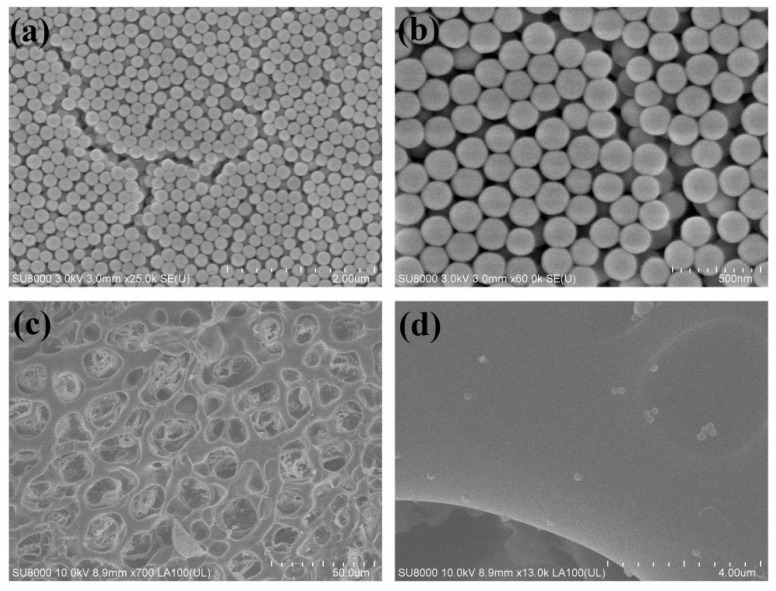
SEM images of the PSBMA-4 nanogel (**a**,**b**) and surface of freeze-dried nanogel-embedded contact lens (**c**,**d**). The scale bars of (**a**–**d**) are 2 μm, 500 nm, 50 μm and 4 μm, respectively.

Compared with emulsion polymerization, reflux-precipitation polymerization is a simple, one-step and surfactant-free method to obtain spherical nanogels with uniform size. The obtained nanogels can be facilely organized and utilized as functional nanobiomaterials for delivery application [36,37,38]. SBMA, which contains a quaternary ammonium group with a positive charge and a sulfo group with a negative charge, was selected as the zwitterionic monomer in this work. Meanwhile, DVB was used as the crosslinker, considering its high reaction activity and strong hydrophobic character. The chemical structure for the prepared PSBMA nanogels was characterized by FTIR spectroscopy. As shown in Figure 1a, two characteristic peaks at 1043 cm^−1^ and 1484 cm^−1^ can be attributed to the symmetric and asymmetric stretching vibrations of the sulfonate groups (–SO_3_) in the structure of SBMA, and the strong peaks at 1719 cm^−1^ and 1179 cm^−1^ are attributed to the stretching vibration of -C=O and -C-O-C-, respectively. In order to study the effect of the monomer/crosslinker feeding ratio on the physicochemical properties of the nanogels, a series of PSBMA nanogels with different cross-linking degrees (the total weight of monomer and crosslinker was 200 mg) were prepared. The colloidal data are shown in Table 1. As the DVB content increased from 10 wt% to 40 wt% (PSBMA-1–PSBMA-4), the diameter of PSBMA nanogels decreased from 294.7 nm to 219.9 nm, and all nanogels displayed narrow size distribution with polydispersity index (PDI) lower than 0.2 (Figure 2). This result was expected because the higher degree of cross-linking led to more compacted nanogels and smaller hydrodynamic sizes [39]. Furthermore, the zeta potential decreased from −4 mV to −14 mV when the cross-linking degree increased from 10 wt% to 40 wt%. The high surface potential was beneficial for colloidal stability. The SEM images of PSBMA-4 nanogels displayed a uniform and spherical morphology, with a calculated diameter of 120–150 nm (Figure 3a,b). It is worth noting that the hydrodynamic size calculated from DLS was larger than that observed by SEM, indicating that the nanogels were highly swollen in aqueous solution and greatly shrunk under dry conditions.

**Figure 4:**
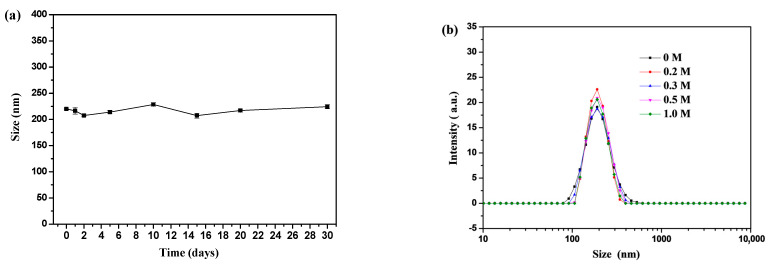
Stability of PSBMA-4 nanogels. (**a**) Changes in particle sizes in water for a month at room temperature. (**b**) The size distributions in different concentrations of aqueous NaCl solution after 24 h incubation at 37 °C.

Nanogels used in biomedicine as drug carriers should have good stability in the physiological environment. As shown in Figure 4a, the PSBMA-4 nanogels displayed good colloidal stability, as the size of particles remained nearly unchanged in water over 30 days, with the change of particle sizes from initial size after storage in the range of ±10 nm, indicating good storage stability. Meanwhile, it was essential to measure the stability of nanogels against salt conditions, as salt exists in humans. In Figure 4b, the nanogels exhibited excellent narrow size distribution, even when the concentration of NaCl increased to 1 M, and the hydrodynamic diameters of nanogels increased only slightly (within 15 nm). The high stability of nanogels can be attributed to their cross-linked structure and the shielding effect of PSBMA zwitterionic chains against the outside environment.

### 3.2. Drug Encapsulation in Nanogels

**Figure 5:**
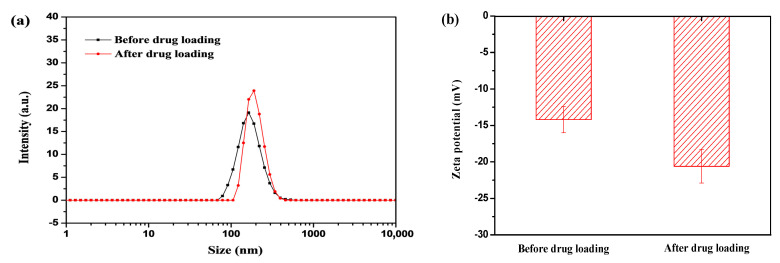
The size distribution (**a**) and zeta potential (**b**) of PSBMA-4 nanogels before and after drug loading.

PSBMA-4 nanogels with the highest cross-linking degree were chosen to conduct the following research because of their small and compact particle size. Meanwhile, the PSBMA nanogels were more hydrophobic because of the higher ratio of the crosslinker DVB, which would have conferred higher LEV loading capability because of the hydrophobic and π-π interactions between LEV and nanogels. The calculated DLC and DLE were 13.3 wt% and 76.8%, respectively, at a theoretical drug feeding ratio of 20 wt%. The size of drug-loaded nanogels increased from 219.9 nm to 267.6 nm (Figure 5a), and the zeta potential decreased from −14.2 mV to −20.0 mV (Figure 5b), further demonstrating successful drug encapsulation into the nanogels.

### 3.3. Synthesis and Characterization of Nanogel-Embedded Contact Lenses

To serve as an ophthalmic drug delivery platform, the drug-loaded PSBMA nanogels were incorporated into a poly(hydroxyethyl methacrylate) (pHEMA) hydrogel matrix (Scheme 1). Briefly, the required quantity of each drug-loaded PSBMA nanogel was dispersed by ultrasonication in a premonomer mixture of HEMA and NVP, a commonly used comonomer in the manufacture of hydrogel contact lenses to boost water content [40]. Then, EGDMA as a crosslinker and AIBN as an initiator were added, and the contact lenses were fabricated by the cast molding method. The formulas for the prepared four batches of nanogel-embedded contact lenses are shown in Table 2, only with the ratio of drug-loaded nanogels varied. pHEMA lenses without nanogels were prepared as a control. The ATR-FTIR spectroscopy of pHEMA contact lens presented in Figure 1b showed typical stretching absorption bands at 1153 cm^−1^ (-C-O-C-), 1708 cm^−1^ (-C=O), 2949 cm^−1^ (CH_2_) and a wide absorption band in 3100−3600 cm^−1^ region relating to the -OH group.

The microstructure for the fabricated nanogel-embedded contact lens was investigated via SEM. The top surface of the freeze-dried pHEMA/LEV-2 contact lens exhibited an interconnecting pore with pore size around 5–25 μm (Figure 3c). The uniform pore size distribution in the microstructure facilitates the diffusion of water molecules across the gel structure. A small number of nanoparticles with spherical shape could be clearly seen in the hydrogel matrix at the given scale, with the diameter around 200 nm (Figure 3d), indicating the successful incorporation of PSBMA nanogels. In addition, data demonstrated that the nanogels were stable and did not break or aggregate during the polymerization process.

**Figure 6:**
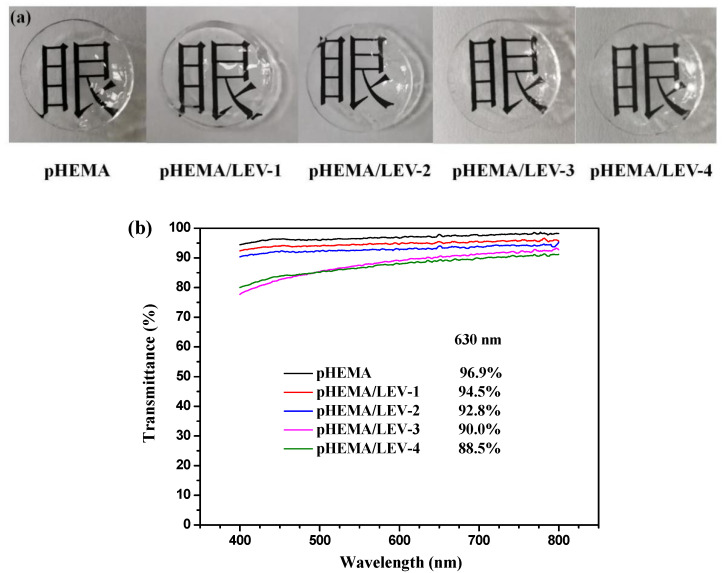
Photographs (**a**) and transmittance spectra (**b**) for the series of nanogel-embedded contact lenses.

The optical clarity of the nanogel-embedded contact lenses was characterized by measuring the light transmittance in the range from 400 nm to 800 nm in a UV spectrophotometer. The pHEMA contact lenses exhibited about 96.9% of transmittance at 630 nm [41]. The transmittances of nanogel-embedded contact lenses decreased as nanogel content increased, and were approximately 94.5%, 92.8%, 90.0% and 88.5% for pHEMA/LEV-1, pHEMA/LEV-2, pHEMA/LEV-3 and pHEMA/LEV-4 at 630 nm, respectively (Figure 6a). The light transparency slightly decreased because of the incorporation of PSBMA nanogels. Similar results were also observed by Xu et al. [27] and Maulvi et al. [28] in that the transmittance of contact lenses decreased when nanoparticles were loaded. The Chinese characters (眼means: eyes) under the series of contact lenses show that optical properties were not affected (Figure 6b), because the incorporated nanogels were monodispersed and were nano-sized (as can be seen from SEM results), which was not likely to significantly affect visual clarity.

**Figure 7:**
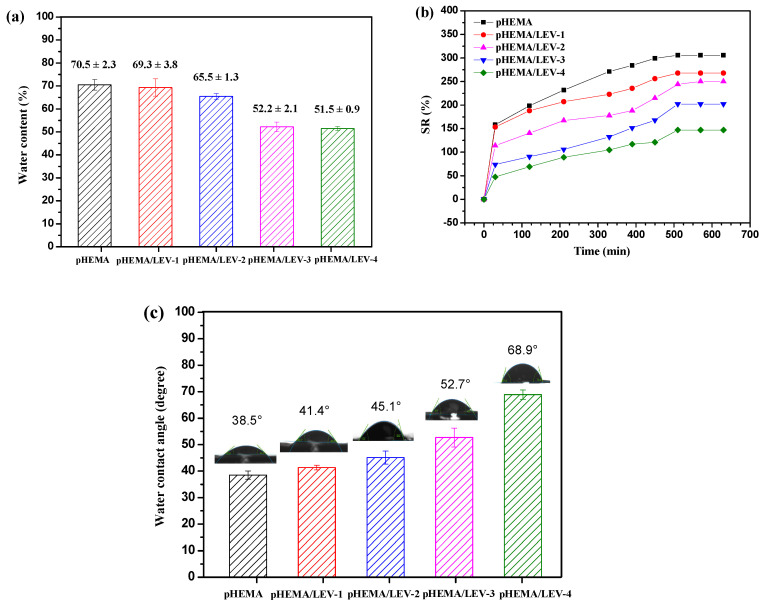
Water content after fully swelling in deionized water (**a**), water contact angle after droplet (5 μL) deposition on the surface of the series of contact lenses at room temperature (*n* = 3) (**b**), and swelling kinetics of contact lenses in water at room temperature (**c**).

The water content of hydrogel contact lenses is a key determinant of their in situ performance and oxygen permeability [42]. In this regard, the EWC was measured by weighting the contact lenses before and after swelling, and the WCA was measured immediately after placing a 5-μL water droplet on the nanogel-embedded contact lenses and on the control pHEMA contact lens surface. Figure 7a shows the EWC (%) values for the series of nanogel-embedded contact lenses and the control pHEMA contact lens. The water content of the pHEMA contact lens was about 70.5%. The very high EWC may be due to the presence of comonomer NVP, which favors the water retention. The EWCs of the series of nanogel-embedded contact lenses were decreased with the increasing nanogel weight ratio, with the approximate results of 69.3%, 65.5%, 52.2% and 51.5%, respectively. The highest incorporation amount with PSBMA nanogels still showed EWC above 50%, higher than many conventional pHEMA-based hydrogels (<50%) [43]. Additionally, the swelling behaviors of the series of contact lenses in water were measured and are shown in Figure 7b. A longer time was needed for contact lenses to reach the equilibrium hydrated state when embedded with the nanogels, compared with pHEMA control, because the water molecules needed to cross the hydrogel matrix to reach the nanogel for swelling to occur. All of the contact lenses reached swelling equilibrium after 9 h, and the equilibrium swelling ratios were in accordance with the EWC measurement before.

Figure 7c shows the WCA for series of contact lenses. The pHEMA hydrogel showed a WCA of 38.5°, indicative of its high hydrophilicity. The pHEMA/LEV series showed a higher WCA than the pHEMA control, while still much lower than 90°, which was indicative of the hydrophobicity that is often considered to be a dominant factor in protein adsorption [44]. The zwitterionic sulfobetaine unit on PSBMA can interact with water via electrostatic forces and hydrogen bonds, which provides a strong water affinity [45]; meanwhile, the porous network of hydrogels also facilitates the retention of large amounts of water. Thus, the incorporation of drug-loaded PSBMA nanogels did not adversely affect the hydrophilicity of contact lenses, suggesting their suitability as a potential material for contact lenses.

**Figure 8:**
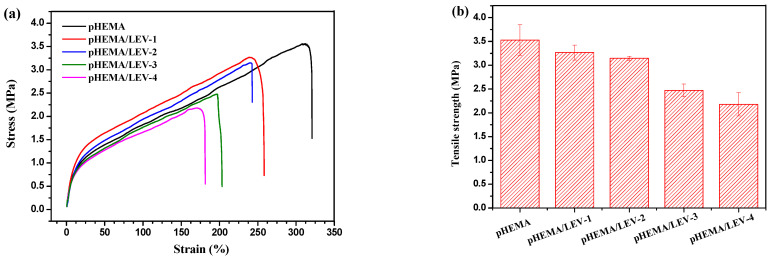
Tensile stress-strain curves (**a**) and tensile strength at break (**b**) of pHEMA hydrogels with different nanogels content.

Good mechanical performance is the basic requirement of contact lenses for sustaining repeated application, removal or eye movement in extended use. The mechanical properties for the series of hydrogels were measured using a tensile tester, and the results are shown in Figure 8. It can be seen that the incorporation of drug-loaded nanogels decreased the mechanical properties slightly. The elongation at break of the hydrogels decreased from 320% to 180%, and the breaking strength decreased from 3.53 MPa to 2.18 MPa. The Young’s moduli calculated based on the slope of the initial linear region in the stress−strain curves for the prepared five sets of contact lenses were in the range of 0.98~1.43 MPa [11,45]. It has been reported that the Young’s modulus of commercial pHEMA-based soft contact lenses is usually in the range from 0.37 to 1.63 MPa. Thus, our prepared hydrogels with appropriate strength and flexibility are suitable to be worn as contact lenses.

To sum up, the loading capacity of the drug-loaded nanogel into a hydrogel matrix should be controlled to obtain optimal wear performance for contact lenses, i.e., the transmittance of the contact lens should be above 90%. Hence, pHEMA/LEV-2 was recommended as a preferred drug-eluting contact lens for the next in vitro drug-release study, considering its sufficient drug-loading capacity and good comprehensive performance.

### 3.4. In Vitro Drug Release of Nanogels and Nanogel-Embedded Contact Lens

**Figure 9:**
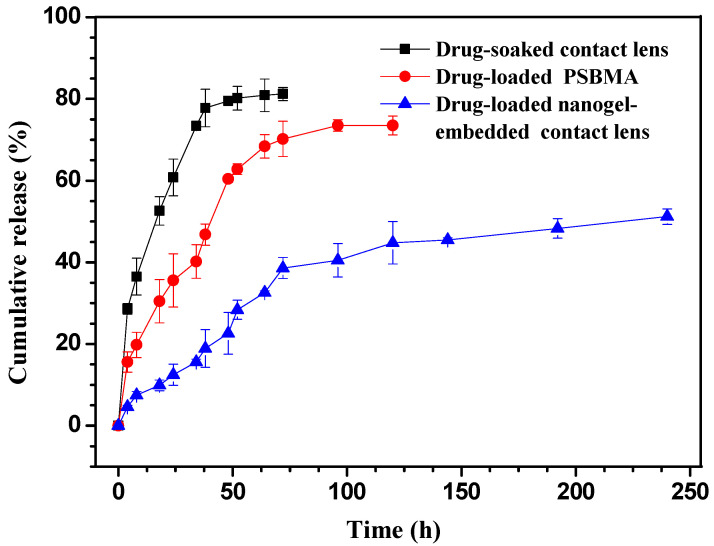
In vitro cumulative release profiles of levofloxacin. (**black**) Free drug-soaked contact lens. (**red**) Drug loaded in PSBMA nanogels. (**blue**) Contact lens embedded with the drug-loaded PSBMA nanogels. (Data were expressed as mean ± SD, *n* = 3).

Before entrapment of the drug-loaded nanogels in the pHEMA lens, the release profiles of the LEV-loaded PSBMA nanogels were studied in PBS (pH = 7.4) at 37 °C using the dialysis bag diffusion method. A known amount of LEV-loaded PSBMA nanogels was placed in a dialysis bag, and the surrounding dissolution medium was sampled at various time points to determine the concentration of LEV via UV-vis. The amount of cumulative drug release was calculated via calibration curves. As seen in Figure 9, initially, the LEV release increased rapidly with time. An initial burst release of approximately 15.6% could be seen in the first 4 h, which may be due to the diffusion of the drug adsorbed on the surface of the nanogels. Drug release continued and reached 73.5% after 96 h. Drug-soaked contact lenses were used as controls. They exhibited a burst LEV release of more than 50% at 18 h, followed by slow release up to 64 h, with a cumulative drug release of 80.9% after 96 h. The fast drug release in the pHEMA hydrogel was mainly driven by physical diffusion due to the drug concentration gradient inside and outside the hydrogel matrix.

As illustrated in Figure 9, the drug-release rate from the nanogel-embedded contact lenses was much slower than that of the drug-loaded nanogels and drug-soaked contact lens. At 3 days, about 22.6% of the drug had been released, and no obvious burst release was detected. Then, the drug was released at a slower but steady pace from day 3 (72 h) to day 10 (240 h) continuously. After 10 days, approximately 55% of the loaded LEV had been released from the contact lenses. The results suggested that the presence of nanogels in the hydrogel further slows down the drug release. The reason is that during the drug-release process, the certain amount of hydrophobic drug which is placed inside the nanogels must first diffuse through the nanogels into the pHEMA hydrogel matrix and subsequently diffuse through the hydrogel.

The amount of LEV loaded in the contact lens was determined by the above LEV loading efficiency (Table 2). Thus, the LEV release from 8% nanogels-loaded hydrogel could be calculated according to cumulative release curve, with approximately 42 μg/day for 10 days on average. The recommended eye-drop dosage for a 0.5% solution of LEV is three drops per day. This means that the daily dose of LEV is calculated at 150 μg (1 drop = 50 μL [46]). Considering that the drug-laden contact lenses have a higher bioavailability (at least 50% [15]) than eye drops, our prepared ophthalmic administration formulation is potentially in the therapeutic range.

### 3.5. In Vitro Cytotoxicity Study

**Figure 10:**
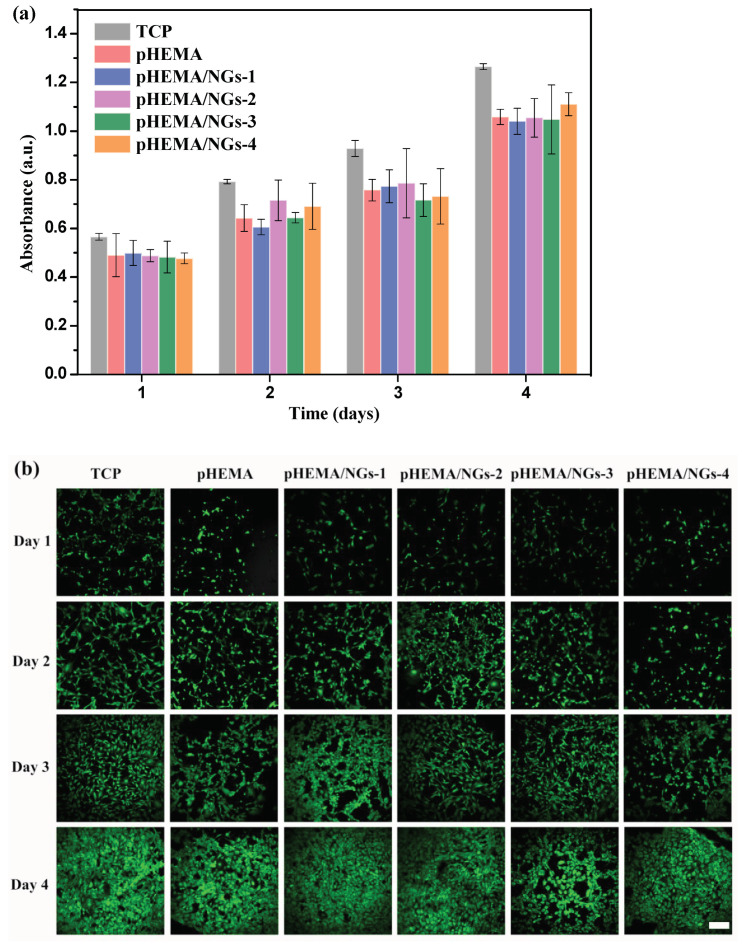
Cytocompatibility test for the series of contact lenses. (**a**) OD values of viable 3T3 cells by CCk-8 assays; error bars represent mean ± SD (*n* = 5). (**b**) Confocal fluorescence images of Calcein AM (green)-stained 3T3 cells incubated on tissue culture plate (TCP) and on the series of contact lenses from day 1 to day 4. The scale bar is 200 μm.

For future commercial applications, the potential toxicity should be taken into careful consideration as the contact lenses can affect the cornea and the surrounding tissue [47]. Biocompatibility was examined by quantitative analysis of cell proliferation activity by CCK-8 assay and observation of cell morphology from confocal micrographs as referred to the literature [48,49,50]. The pHEMA contact lens and series of nanogel-embedded contact lenses without drug loading (pHEMA/NGs) were incubated with mouse embryo fibroblast (3T3) cell lines for between 1 and 4 days, and the calculated OD (absorbance at 450 nm) of cell cultures was in direct proportion to the numbers of viable cells. Cells cultured on TCP were used as the negative control. As shown in Figure 10a, the OD values of cells cultured onto each contact lens showed a significant increase from day 1 to day 4, with only a small gap with native control TCP, indicating that the cell proliferation activity was not significantly affected by the introduction of contact lenses. Furthermore, the OD values of cells cultured on nanogel-embedded contact lenses (pHEMA/NGs) showed no obvious difference from that of pHEMA contact lenses. The results illustrate that the embedded nanogels did not significantly influence cell viability either, in agreement with the biocompatibility of PSBMA nanogels reported in the literature [37].

Furthermore, the morphologies of 3T3 cells grown on contact lenses for 1, 2, 3 and 4 days were observed through CLSM after staining with green fluorescence calcein-AM, with TCP as negative control. As shown in Figure 10b, the growth situation and growth velocity of 3T3 cells displayed no obvious difference when the cells were incubated on different contact lenses, which was in accordance with the CCK-8 cell proliferation results. The 4th-day growth conditions of all 3T3 cell groups displayed a regular spindle phenotypic shape, which was similar to the nature of the morphology of fibroblasts cultured on TCP. Hence, our prepared nanogel-embedded contact lenses possess good biocompatibility, verifying their promising application as drug delivery systems.

## 4. Conclusions

In summary, we have developed novel nanogel-embedded contact lenses for extended ophthalmic drug delivery. First, a series of PSBMA nanogels were synthesized by the reflux-precipitation polymerization method, and their uniform spherical morphologies and good colloidal stability were demonstrated by SEM and DLS. Ophthalmic LEV was then incorporated into the nanogels with high drug-loading capacity. Nanogel-embedded contact lenses were obtained by the cast molding method after mixing the drug-loaded nanogels into premonomer solution. The prepared hydrogels exhibit a closed-type porous structure, and spherical nanogels could be clearly seen dispersed in the hydrogel matrix. The evaluation of light transmittance, WCA, EWC, swelling behavior and mechanical properties of the series of nanogel-embedded contact lenses revealed that with an increase in embedded nanogels, the critical quality of the lens decreased slightly. However, the key properties of the optimized contact lens (pHEMA/LEV-2) with moderate incorporation of nanogels (8 wt%) remained within the range of values recommended for contact lenses, with light transmittance above 90% and EWC of 65.5%, etc. In vitro drug release revealed sustained LEV release in the therapeutic range for 10 days with no initial burst release. Furthermore, a cell viability assay and cell morphology observations demonstrated the cytocompatibility of the prepared contact lenses. In conclusion, our prepared nanogel-embedded contact lenses have great potential to serve as novel ophthalmic drug delivery systems.

## Figures and Tables

**Table 1:** Colloidal properties for the series of PSBMA nanogels with different monomer/crosslinker feeding ratios.

Sample	SBMA (mg)	DVB (mg)	AIBN (mg)	AN (mL)	Size (nm) ^a^	PDI ^b^	ZP (mV) ^c^
PSBMA-1	180	20	4	40	294.7 ± 2.6	0.183	−4.3 ± 0.7
PSBMA-2	160	40	4	40	270.4 ± 3.6	0.124	−9.2 ± 1.3
PSBMA-3	140	60	4	40	248.6 ± 2.2	0.164	−11.4 ± 1.5
PSBMA-4	120	80	4	40	219.9 ± 1.7	0.104	−14.2 ± 1.8

Notes: (^a^) Measured in phosphate buffer of pH 7.4 at 25 °C (1 mg mL^−1^); (^b^) Polydispersity index of the particle size; (^c^) Zeta potential of nanogels in phosphate buffer of pH 7.4 at 25 °C (1 mg mL^−1^).

**Table 2:** The formulas for the series of nanogel-embedded contact lenses.

Sample	NVP (mg)	HEMA (mg)	EGDMA (mg)	AIBN (mg)	Drug-Loaded Nanogels (mg)	Loaded LEV (mg) ^a^
pHEMA	150	815	15	20	0	0
pHEMA/LEV-1	150	815	15	20	50	6.7
pHEMA/LEV-2	150	815	15	20	80	10.6
pHEMA/LEV-3	150	815	15	20	110	14.6
pHEMA/LEV-4	150	815	15	20	130	17.3

Notes: (^a^) Calculated by drug loading content of nanogels.
